# Gestures for Picture Archiving and Communication Systems (PACS) operation in the operating room: Is there any standard?

**DOI:** 10.1371/journal.pone.0198092

**Published:** 2018-06-12

**Authors:** Naveen Madapana, Glebys Gonzalez, Richard Rodgers, Lingsong Zhang, Juan P. Wachs

**Affiliations:** 1 School of Industrial Engineering, Purdue University, West Lafayette, Indiana, United States of America; 2 Goodman Campbell Brain and Spine, Indianapolis, Indiana, United States of America; 3 Department of Statistics, Purdue University, West Lafayette, Indiana, United States of America; University of Groningen, University Medical Center Groningen, NETHERLANDS

## Abstract

**Objective:**

Gestural interfaces allow accessing and manipulating Electronic Medical Records (EMR) in hospitals while keeping a complete sterile environment. Particularly, in the Operating Room (OR), these interfaces enable surgeons to browse Picture Archiving and Communication System (PACS) without the need of delegating functions to the surgical staff. Existing gesture based medical interfaces rely on a suboptimal and an arbitrary small set of gestures that are mapped to a few commands available in PACS software. The objective of this work is to discuss a method to determine the most suitable set of gestures based on surgeon’s acceptability. To achieve this goal, the paper introduces two key innovations: (a) a novel methodology to incorporate gestures’ semantic properties into the agreement analysis, and (b) a new agreement metric to determine the most suitable gesture set for a PACS.

**Materials and methods:**

Three neurosurgical diagnostic tasks were conducted by nine neurosurgeons. The set of commands and gesture lexicons were determined using a Wizard of Oz paradigm. The gestures were decomposed into a set of 55 semantic properties based on the motion trajectory, orientation and pose of the surgeons’ hands and their ground truth values were manually annotated. Finally, a new agreement metric was developed, using the known *Jaccard* similarity to measure consensus between users over a gesture set.

**Results:**

A set of 34 PACS commands were found to be a sufficient number of actions for PACS manipulation. In addition, it was found that there is a level of agreement of 0.29 among the surgeons over the gestures found. Two statistical tests including paired t-test and Mann Whitney Wilcoxon test were conducted between the proposed metric and the traditional agreement metric. It was found that the agreement values computed using the former metric are significantly higher (*p* < 0.001) for both tests.

**Conclusions:**

This study reveals that the level of agreement among surgeons over the best gestures for PACS operation is higher than the previously reported metric (0.29 vs 0.13). This observation is based on the fact that the agreement focuses on main features of the gestures rather than the gestures themselves. The level of agreement is not very high, yet indicates a majority preference, and is better than using gestures based on authoritarian or arbitrary approaches. The methods described in this paper provide a guiding framework for the design of future gesture based PACS systems for the OR.

## Introduction

With the introduction to Electronic Medical Records (EMR) and Electronic Health Records (EHR) into hospitals by the end of the nineties [1–4], the traditional human-computer interfaces (HCI) paradigm (including the mouse and keyboard), became the standard by default. It took about ten years to notice the first reported studies linking touch based interfaces to the spread of nosocomial infections [5–11]. While some solutions have been suggested to address this issue, such as using disinfectants to decontaminate the keyboards [6, 8] and using sealing materials to protect the mice and screens [9, 12, 13] it was found that surgeons ended up delegating operational instructions to the nurses outside the aseptic area [14]. Instead, gesture based interaction became the most attractive solution since it allowed the surgeons to control Picture Archiving and Communication System (PACS) by themselves without moving from the patient’s side. As opposed to traditional interfaces where there is consensus on the specific “metaphors” to trigger menu options (e.g. click, drag, move the mouse), with gestures there is not a clear consensus about the gestures’ functions [15–17]. Under these circumstances, the optimal gestures can be determined arbitrary by the designer (authoritarian approach), or alternatively, based on those gestures that will be easy to “recognize” by the sensor attached to the PACS [15, 17, 18] (technology driven approach). In either way, if the surgeons need to only “navigate” MRI datasets on the PACS, four cardinal functions would be enough (seven if including zoom-in/out and selection) and any solution involving more elaborate procedures may be considered an overkill. Unfortunately, a user task analysis on PACS systems’ revealed that software such as Synapse, OsiriX, or OnePacs offer at least a hundred functions for image manipulation [19–21]. Yet, it would be impractical to use all the possible functions since users can only remember only a small set of those gestures due to humans’ cognitive capacity [22]. In such context, any research study proposing effective ways to determine the best gestures to operate such application is of paramount importance.

To tackle this hurdle, the “consensus” approach has been proposed. In this approach, the set of gestures is chosen based on the amount of agreement among the users (e.g. surgeons) [23–28]. It is known from speech, hearing and language studies (SHLS) that most people agree on very few gestures: 70% of the people agree on only 30% of the gestures [29]. It has been speculated that within a certain domain of expertise, agreement levels would increase. One of the objectives of this paper is to validate or refute that claim, and alternatively study whether some key components of the gestures are more “popular” than others among surgeons. Such analysis requires decomposing the gestures into basic factors (semantic descriptors) and thereby requires new metrics for agreement analysis, which is another contribution of this paper.

To summarize, the main contributions of this work are: (1) determine the most common set of functions required to operate a PACS in the OR—such function set will determine the number of gestures required; (2) a new index to measure agreement among domain experts; (3) an agreement value on user selected gestures.

The paper is organized as follows: Section (II) will present the state-of-the-art regarding gesture based interfaces and gesture selection procedures; Section (III) will introduce initial findings based on empirical studies and the method proposed to measure agreement; Section (IV) will present results of the methodology, and Section (V) will present a discussion and conclude the work.

## Background

In the last ten years, hand gesture based interaction systems for PACS control have been introduced in the OR in order to reduce the risks of diseases spread through direct contact [30]. Most of the research in this field focused on algorithms and sensors enhancement [16, 17, 26, 31–33]. Such studies assumed a smaller and constrained set of commands (less than 20 gestures) for PACS operation, in order to relax the complexity of the systems.

In order to maintain sterility in the OR, it is common practice for a surgeon to convey the image manipulation commands verbally to an assistant who is sitting near the computer and operating the PACS [34]. However, studies show that this approach involves verbal miscommunications that lead to significant delays in the surgical procedures [14, 35, 36]. Recently, gesture based interaction was compared against direct manipulation with a mouse and against verbal transfer of information to an assistant [36]. Their results show that the gesture modality is significantly more efficient than verbalizing the instructions.

Multimodal systems have also been explored in the area of automatic recognition in the OR. Some of these studies focus on interfaces that allow both voice and gestural commands [37]. Particularly, the work by [38] aims to design an architecture that would allow natural gestures only, leaving all the other actions to voice recognition. The work in [39] expands the multimodal concept by adding an autonomous modality, where the system determines the actions to assist the surgeon based on the contextual information [36]. Alternatively, gesture design from the usability front was studied in [39, 40]. For example, the work in [14, 30], explores the socio-technical aspects that constrain a gestural interface in the OR. Additionally, [22] studied the memorability of gestural interfaces by comparing random, pre-designed and user-defined lexicons. The results of this work show that a user can recall 15-16 gestures with a very little learning time if they are customized.

Agreements studies are very common first step when deciding on a lexicon for a gestural interface. Agreement analysis are also a common part of what is referred as elicitation studies [25, 26, 29, 41]. The agreement found tends to vary greatly according to the number of commands, the type of interface, number of subjects and the way that similar commands are grouped together [26, 42]. In addition, some studies have tried different grouping taxonomies to determine the properties in gestures that give a higher consensus. In [42] the gestures were divided into symbolic actions (0.35 agreement) and direct manipulation (0.18 agreement). In [43], the gestures were classified as: metaphorical, symbolic, physical and abstract. [25, 27] came up with a taxonomy to describe and classify their gestures and measure the consensus according to those properties. Other user consensus works are not limited to the “2D” manipulation of the space, in [24] an elicitation study for augmented reality tools was conducted, finding a 29% agreement between subjects.

The vast majority of elicitation studies use the metric provided by [27]. That metric does not correlate clearly to the percentage of the population that agrees on a gesture. The work in [29] assesses agreement between subjects using a different metric, that is more similar to the Jaccard index [44]. This study revealed that in general, most user elicited lexicons follow a 70 : 30 rule, where 70% of the subjects agree on 30% of the commands.

## Methodology

This study was done with the approval of the Institutional Review Board (IRB) of Purdue University. Protocol number: 1606017821. The aim of this work was to determine the gestures that surgeons prefer for controlling a PACS in the OR. The process followed consisted of three main steps: 1. *Command extraction*, 2. *Unconstrained gesture elicitation* and 3. *Agreement analysis*. In the command extraction step, neurosurgeons were observed while performing three typical diagnostic tasks using Synapse (a radiology image browser). Based on this observational study, the most common PACS functions were determined. In the second part, an unconstrained gesture elicitation study was conducted to elicit natural gestures from surgeons following a Wizard of Oz paradigm [45]. Lastly, an *Agreement analysis* was performed using a novel approach that incorporates the properties of the gestures. The following sections illustrate these three steps in more detail.

### Participants

Nine neurosurgeons with one-two years of experience in operating PACS were recruited on a voluntary basis. The study was conducted at Goodman Campbell Brain and Spine, Indianapolis, which is a part of the Indiana University School of Medicine (IUSM). Written informed consent was obtained from all subjects.

### Step 1. Command extraction

**Fig 1:**
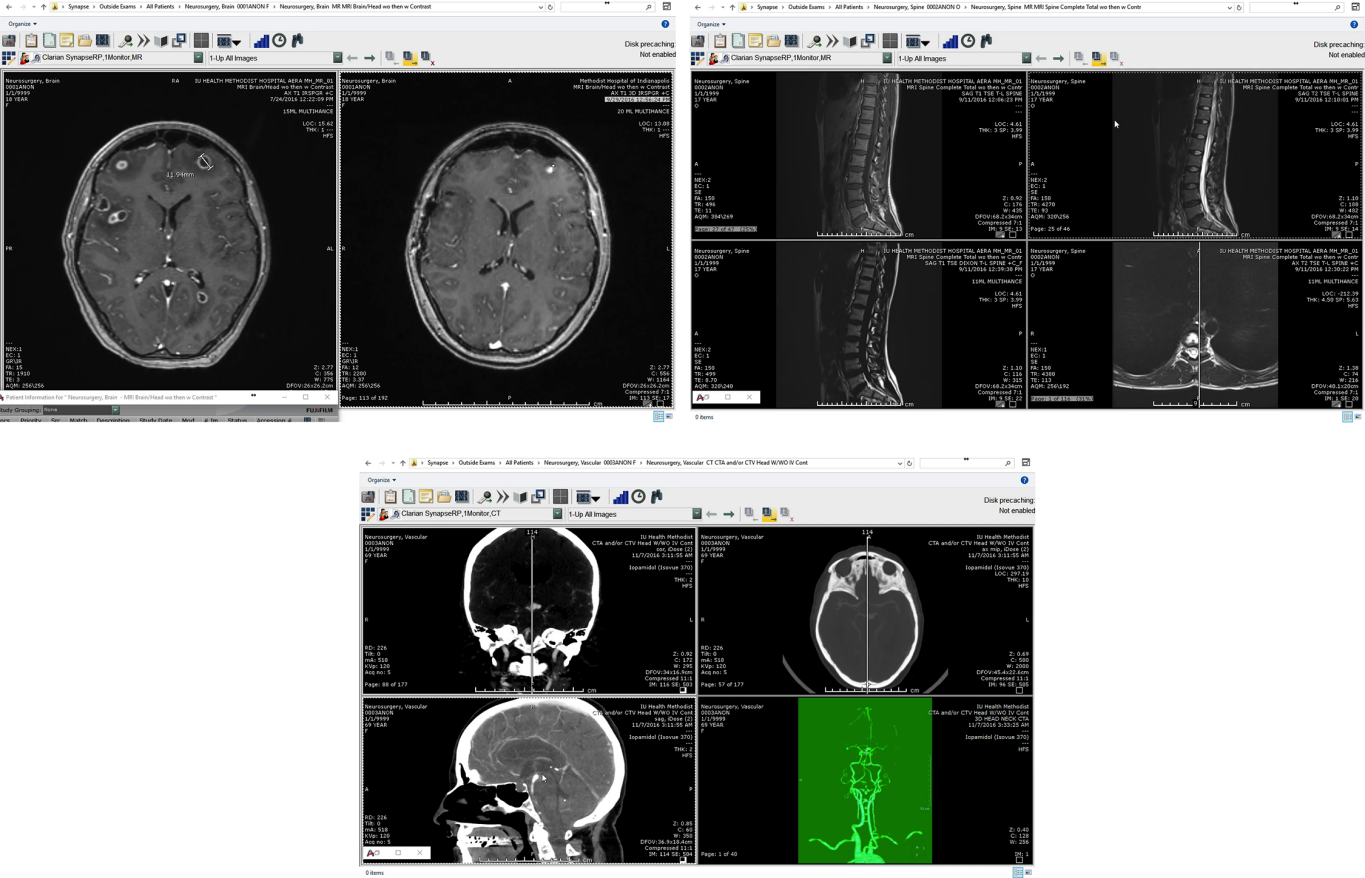
Snapshots taken during each task using Synapse. *Upper left (Task 1):* Comparing two bifrontal lesions. *Upper right (Task 2)*: Display the same spine lesion in different cuts. *Center (Task 3)*: Locate and display a basilar aneurysm in 2D and 3D sequences.

Synapse^®^ software is widely used at IUSM for browsing radiology images. It complies with the DICOM standard to store the medical images and to integrate the imaging devices such as MRI scanners with the PACS system. Three medical image manipulation tasks encompassing most of the functionalities of Synapse^®^ were considered. These tasks were chosen by expert neurosurgeons since they are diagnostic tasks that are routinely performed in the operating room. Each task has a set of well-defined goals such as identifying and displaying lesions in different image sequences of brain or spine scans. The tasks included: T1. Comparing and measuring bi-frontal lesions from two different studies of a brain MRI, T2. Finding and displaying a spinal lesion in three different MRI sequences, and T3. Displaying a basilar aneurysm in a brain CT scan and manipulate a 3D sequence for the same aneurysm. Fig 1 shows some screen-shots of these tasks.

Initially, each neurosurgeon was asked to accomplish these tasks using keyboard and mouse interfaces. By tracking the menu choices, mouse motions and selections, all functions required to complete the tasks using the PACS system were collected. At the end of the study, the subjects had the option to suggest additional commands that they would have used as alternatives to the ones suggested in the tasks. From over a hundred functions found to be available in Synapse^®^, the surgeons used only a subset of 34 functions to accomplish the tasks. The additional feedback confirms that these functions are sufficient for the vast majority of procedures conducted at the hospital.

The obtained commands were grouped into meaningful clusters based on semantic and numerical similarity. This allowed to reduce the number of gestures that surgeons would need to remember. We adopted a context based approach where the same gesture would mean different things based on the context used. This gesture based interface’s architecture consists of two layers: *context* and *modifier*. The *context* represents the general action that the surgeon wants to perform, i.e. Zoom, Pan, Scroll, and the *modifier* represents the different options for that command, i.e. *In* and *Out* for *Zoom*, or *Up*, *Down*, *Left* and *Right* for *Pan* and *Switch Panel*.

### Step 2. Gesture elicitation

For this part of the study, the neurosurgeons were initially required to design a lexicon for controlling the medical imaging software. For each of the 34 commands, the surgeons proposed a gesture. When the gestures belonged to different layers (*context* and *modifier*), reutilization of the gesture was allowed. Then, a completely randomized statistical design was used to assign the surgeons to one of the three tasks (T1, T2 and T3) mentioned in the previous section. The subjects then proceeded to complete the task by using their designed gestures in a Wizard of Oz setting [45, 46]. This arrangement consisted of two researchers sitting behind a screen and simulating to be the automatic gesture recognition system. The gesture performed by the participant (surgeon in this case) was recognized and interpreted by one of the researchers who controls the PACS system. The gestures elicited during the Wizard of Oz experiment were recorded using a Kinect^™^ camera.

**Fig 2:**
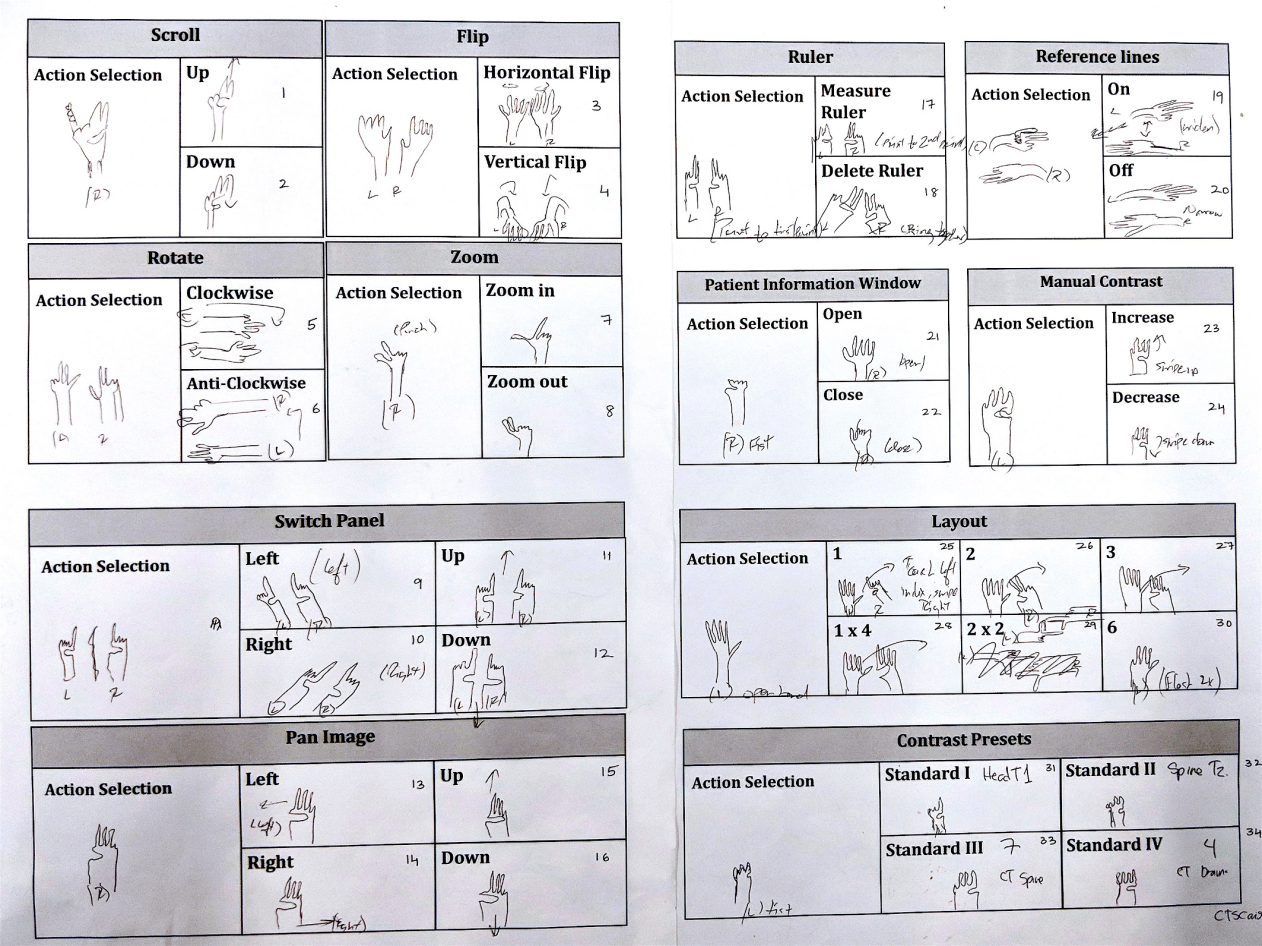
This form contains a list of 34 commands. Each command is highlighted in gray. The rectangle at the left of the command corresponds to the context of the gesture and the 2-4 rectangles to the right correspond to the modifiers.

The *gesture elicitation* step was done on a drawing board, where the surgeons are required to draw the gestures that they prefer for each of the 34 commands of PACS (see Fig 2). For each gesture group (gestures that share similar context), the surgeon had the chance to design a common gesture for context (action selection) and a unique gesture for the modifier. This software design architecture allowed reusing the modifier gestures that belonged to different contexts.

After the gesture design step, the neurosurgeons were required to complete one of the three diagnostic tasks using the Wizard of Oz paradigm [45]. They were asked to use the gestural lexicon that they previously designed to interact with the software. The result from this phase was 9 gesture lexicons. Each one of them containing 34 commands. Thus, a total of 306 (34 × 9) gestures was collected.

### Step 3. Agreement study

Agreement metrics measure the extent to which surgeons agree on a specific gesture for a given command. In this study, the gestures obtained in Step 2 were used to assess the level of agreement among surgeons. The level of agreement was measured using two methods: Method 1. The metric proposed by [27] and Method 2. A metric proposed in this work which is based on a novel representation of gestures using semantic descriptors [47]. The following sections explain both metrics in detail.

#### Method 1

The state-of-the-art approach proposed by [27] is widely used in the literature to measure the level of consensus among subjects. This method has two stages: grouping and evaluation. In the grouping stage, the gestures for the same command were clustered by similarity through visual inspection. Thus, all the gestures that are placed in the same group are considered equal. Then, the consensus for each command is obtained by summing the squares of the size of each group, divided by the total number of gestures available for that command. This metric takes a value of one when there is complete agreement (consensus) i.e. all of the gestures chosen for a command were identical and a value of 1 over the total number of gestures $\left(\frac{1}{N}\right)$ for that command when there is no agreement [27].

There are two major problems with this approach. First, the agreement value is non-zero when there is no agreement at all. Second, the literature does not provide a good qualitative interpretation of this metric. For example, if an agreement of 0.20 is found, this does not mean that 20% of the group agrees on a gesture. Thus, this metric is of little use when looking for an optimal lexicon to control a medical software. To compensate for the limitations of the state of the art approach, a novel method to measure the level of agreement is proposed and explained in the next section.

#### Method 2

Previous efforts to find the level of agreement considered the gesture as a concrete entity, ignoring their embedded properties (e.g. hand shape, hand motion trajectory, plane of motion, etc.). This work proposes to represent each gesture as a combination of semantic descriptors [47] and compute the level of agreement for each command. Table 1 describes the complete list of 55 semantic descriptors. The properties are grouped by category for easier visualization.

**Table 1:** The complete set of 55 semantic descriptors, grouped by category.

#	Category	Semantic descriptor
1	Hand Motion (for both dominant and non-dominant hand)	Right, Up, Left, Down, Forward, Backward, Clockwise, Counter-clockwise, Iterative, Circle and Rectangle.
2	Combined movement	Circle and Rectangle.
3	Overall flow	Inward and Outward.
4	Orientation of the hand (for both dominant and non-dominant hand)	Right, Up, Left, Down, Forward, Backward.
5	Orientation of the hand (for both dominant and non-dominant hand)	Closed-0 fingers, 1 finger, 2 fingers, 3 fingers, 4 fingers, Open-5 fingers, C shape, V shape
6	Shifts 1	Palm/Dorsal shift

The main idea of this approach lies in representing a gesture as a binary vector, where each element of the vector is a semantic property. If the property is present in the gesture, then the value for that element is one, otherwise the value is zero. Initially, each of the 306 gestures are decomposed into two gestures: a context gesture and a modifier gesture, as described at the end of step 1. Then, the context and the modifier are manually annotated resulting in a sparse binary vector for each gesture.

To measure the similarity between two gestures, the well known Jaccard metric *J* was used in the same way as in [44, 48] This metric is a suitable method to evaluate the distance between two sparse binary vectors. The overall agreement with Jaccard can be calculated using the equation below. The agreement per command is defined as the average Jaccard similarity of all possible pairs of gestures corresponding to this command. The overall agreement is defined as the mean agreement among the 34 commands. In this formulation (see Eq (1)) *N*_*c*_ represents the total number of commands and *N*_*g*_ represents the total number of gestures per command. *S*_*i*_ and *S*_*k*_ represent the gesture examples *i* and *j* for the command *k*.

$$\begin{array}{c} A_{overall}=\frac{2}{N_{c}N_{g}\left(N_{g}-1\right)}\sum_{k=1}^{N_{c}}\sum_{i=1}^{N_{g}}\sum_{j=i+1}^{N_{g}}J\left(S_{i}^{k},S_{j}^{k}\right). \end{array}$$ (1)

The next section shows the results of the agreement analysis using Metric I and Metric II. In addition to assessing the agreement for the full gesture (context and modifier combined), we also study the agreement on the context and modifier individually.

## Results and discussion

**Fig 3:**
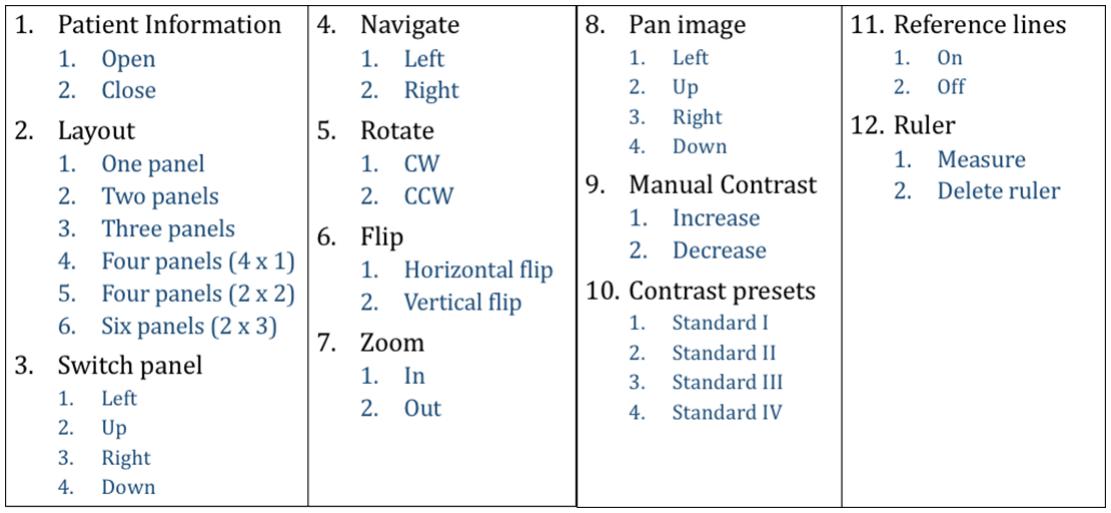
The complete set of commands that surgeons require to fully operate PACS in the OR.

The goal of the command extraction phase was to find the subset of PACS actions that neurosurgeons use when querying patient information or manipulating images in the OR. The Synapse^®^ [20] software offers over a hundred possible commands. Nevertheless, the extraction process revealed that only 34 of those commands are used in the OR as shown in Fig 3. Further, the commands are grouped by “context” (1 to 12) and each context has two to four “modifiers”. These actions are also available in other popular PACS such as OsiriX^™^ [21] or OnePacs^™^ [19]. Thus, the commands found in this study can be used as a standard set in any other medical information system that has a PACS. Since the number of commands is too big for a completely customized interface [22], an agreement study is crucial for a good gestural interface design.

An agreement analysis was conducted using both the traditional method (Metric I) and our approach based on semantic descriptors (Metric II). The results are shown in Table 2. This table shows the agreement index for the full gesture (context + modifier), context only, and modifier only. The reason for splitting the results into these three categories lays on the fact that the command’s context tends to be more abstract (i.e. open Patient Information Window, change layout, select contrast, etc.), while the modifier tends to have direct effect on the image (i.e. up, down, left, right, zoom in, zoom out). Results show that the level of agreement on the context’s gestures is much lower in comparison to the modifier’s gestures, justifying the claim that context gestures are abstract and show large variance between the subjects. The highest agreement index was found with the modifier gestures, with a value of 0.34 with the Metric II as shown in Table 2. A one tailed, paired t-test (*DOF* = 34 − 1 = 33, *α* = 0.05) was performed to ensure that the difference between the metrics was statistically significant. The null hypothesis stated that the agreement indices for Metric I and Metric II were the same, while the alternative hypothesis stated that the index of Metric II was greater than the index of Metric I. The difference between methods was statistically significant (*p* < 0.05) for the modifier and the context + modifier. The context did not show a significant difference between the metrics, since it was equally low in both cases. It is a known fact that the t-tests are suitable for normally distributed data. However, the distribution of the agreement data is not guaranteed to satisfy that condition. In order to test the robustness of these results, an additional Mann Whitney Wilcoxon test was conducted. This test corroborated the statistical significance of the previous results.

**Table 2:** Consensus measured by the Metric I (*State of the art*) and Metric II (*The Jaccard distance using semantic descriptors*).

*Category*	*Metric I(SOA)*	Metric II (SDs)
Context	0.13 ± 0.02	0.1146 ± 0.09
Modifier	0.23 ± 0.13	**0.34 ± 0.14** *
Context + Modifier	0.13 ± 0.02	0.29 ± 0.06*

* = statistically significant, *p* < 0.05.

Metric I considers gestures as hard entities, ignoring the common semantic properties in dissimilar gestures. Hence, it resulted in a lower agreement measure, especially for the unconstrained gesture elicitation studies. This contradicts the intuition that agreement among subjects coming from the same domain is higher than 30% for 70% of the subjects [29]. In contrast, Metric II represents the gesture as a combination of semantic properties accounting for the similarities in the dissimilar gestures. Metric II yields a higher agreement, because it captures the fact that the subjects may not agree on the complete gesture but might agree on some high-level properties of the gesture.

**Fig 4:**
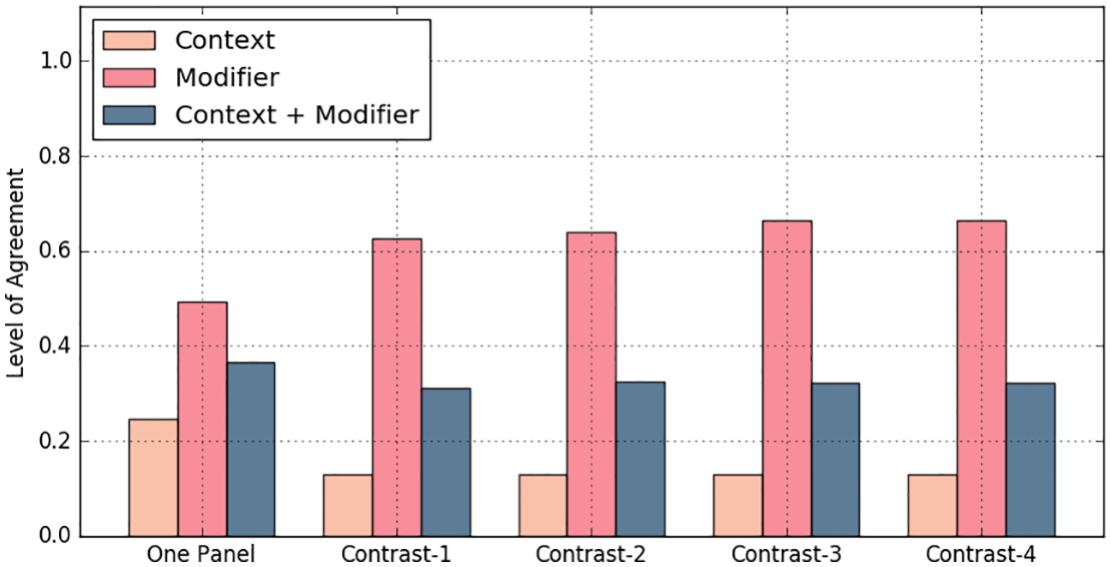
Consensus of the top five gestures with highest level of agreement.

Fig 4 shows the top 5 gestures with the highest agreement index using the Semantic Descriptors approach. Again, the modifier is the part of the gesture that shows the highest level of agreement. These commands involve gesturing a number in the modifier. It means that the surgeons have a standardized way of gesturing the numbers from one to four.

A complete set of guidelines for selecting optimal gestures can be easily extracted from the common semantic properties of the elicited gestures. First, the overall semantic representation for each command is found by averaging the semantic vectors of individual gestures corresponding to a command. There are nine gestures per command in this study. The semantic descriptors (elements in the semantic vector) that have the highest values would represent the gestural properties with the highest agreement. For example, in the case of zoom, 70% of the participants chose a gesture with an inward flow for zoom out and with an outward flow for zoom in. This means that the most important principle when it comes to designing a zoom gesture is the inward or outward movement. This process is repeated across the complete set of commands and a set of guidelines/principles for each command is constructed based on the semantic properties preferred by the majority. Further, an optimal gesture lexicon is obtained by choosing the gesture that had the highest agreement index, according to the Metric II. It has to be ensured that the gestures corresponding to the PACS commands should abide by these guidelines in order to achieve an optimal gesture lexicon.

## Conclusion

In recent years, there has been a major spur of hand-gesture interfaces for controlling Electronic Medical Records in the Operating Room. Yet, it is still not clear which gestures should be used to operate these interfaces. This work addresses the challenge of determining the best gestures to control a PACS system in the OR, based uniquely on agreement among surgeons. Previous work found that most people agree on a very small set of gestures and therefore gestures can be arbitrarily selected. However, this paper makes a more careful observation of how agreement is measured and proposes a new metric based on the gesture components rather than the gestures themselves. It is reported that there is a significant agreement among surgeons on the key components of gestures (level of agreement of 0.29), as opposed to previously reported values (below 0.13). Such results imply that the proposed agreement index (Metric II) could be used as the main criteria for gesture selection in such interfaces. Other contributions of this paper include: 1. Determining the number of commands required to manipulate a PACS software effectively; 2. Identifying what are the gestures that are preferred by surgeons; 3. Determining the level of agreement among surgeons on the aforementioned set of gestures. Based on the level of agreement of the gestures’ components the system designer can select those gestures associated with the most popular components; or alternatively learn a set of heuristics that can serve as a guiding criterion for the design of future gesture based interaction systems in the OR.
